# Immune Delay, Beyond Immune Evasion, as a Driver of Pathogen Propagation Competence Through Neutrophil Dysregulation, to be Mitigated by Low-Frequency Electromagnetic Fields (LF-EMF)

**DOI:** 10.3390/ijms27010143

**Published:** 2025-12-23

**Authors:** Jan J. M. Cuppen, Huub F. J. Savelkoul

**Affiliations:** 1 Cell Biology and Immunology Group, Wageningen University and Research, 6708 PB Wageningen, The Netherlands; huub.savelkoul@wur.nl; 2 Neiding BV, Werfberg 12, 5508 HB Veldhoven, The Netherlands

**Keywords:** electromagnetic fields, neutrophils, infection, immune delay, immunomodulation

## Abstract

This paper proposes that immune delay, beyond immune evasion, is key in the propagation competence of major viral and bacterial infections, and that the dynamics of infection and immune response suggest possibilities for mitigating the ensuing infectious diseases. Recent data show a critical role of neutrophils at various stages of viral and bacterial infections, revealing how early activation of neutrophils could help mitigate infectious diseases. It could prevent the gradual overactivation of subclasses of neutrophils and probably not induce it. In respiratory virus infections, an immune delay of several days allows the development of a high viral load supporting infectivity towards further hosts when a delayed and increased immune response takes place. Virus variants will optimize immune delay towards highest infectivity, supporting pandemic potential. The influenza virus, coronavirus, and several major bacterial infections exhibit such immune delay capability. Recurrent urinary tract infections (rUTI) are common, often associated with the causative uropathogenic *E. coli* (UPEC) that has this capability, suggesting that immune delay is crucial in the pathogenesis of rUTI and other widespread bacterial infections. Counteracting immune delay, therefore, is a promising approach for mitigating infectious diseases with epidemic and pandemic presence or potential. Previously proven low-frequency electromagnetic field (LF-EMF)-induced neutrophil activation is such an approach.

## 1. Immune Evasion

Immune evasion refers to the ability of pathogens to avoid or subvert the induction of an effective host immune response, thereby allowing them to persist and cause overt disease [1]. This is a critical aspect in many infectious diseases, as it enables pathogens to establish long-term infections and evade clearance by the immune system. Bacterial immune evasion to circumvent host immune defenses represents a collection of molecular strategies that, in the evolutionary model, have evolved as a consequence of host–pathogen coevolution. Through surface masking, complement inhibition, intracellular survival, and biofilm formation, bacteria have developed different and overlapping strategies targeting immune recognition and protective effector functions.

Viruses and their hosts are engaged in a continuous evolutionary arms race in which immune pressures select for viral variants with an enhanced capacity to evade detection and immune-mediated viral clearance. The host innate immune system, through pattern recognition receptors (PRRs, see Table 1) such as RIG-I-like receptors and Toll-like receptors, detects viral nucleic acids and induces type I interferon (IFN) responses, while adaptive immunity results in the formation of neutralizing antibodies and the activation of cytotoxic T lymphocytes to eliminate infected cells. In turn, viruses evolved various immune evasion mechanisms, including shielding viral RNA from detection, blocking IFN induction and subsequent signaling, downregulating MHC (Major Histocompatibility Complex) class I to avoid CD8^+^ T-cell recognition, and altering surface expression of viral antigens through mutation, recombination, or glycan shielding to escape antibody binding. Persistently present viruses further employ latency, integration, or modulation of cell death pathways to minimize their immunogenicity. These viral strategies drive reciprocal host adaptations, including diversification of PRRs, rapid evolution of antiviral restriction factors, and variation in MHC polymorphisms at the population level, all resulting in a dynamic equilibrium without a unilateral complete dominance. This co-evolution drives the diversity of viral pathogenesis and the continuous emergence of immune-escape variants across many viral families [2,3,4,5,6]. In addition, the evolutionary arms race between bacterial pathogens and host immune systems has produced an extraordinary diversity of immune evasion strategies. While each bacterial family has developed unique molecular solutions that uniquely evade the ensuing immune response, several common strategies emerge from comparative analysis: surface modifications to avoid recognition, neutralization of complement and antimicrobial peptides, manipulation of phagocytic cells, and establishing protected niches [7,8]. Bacterial lineages repeatedly converge on masking pathogen-associated molecular patterns (PAMPs) with host-like glycans causing molecular mimicry, and on targeting neutrophil function [9,10]. Horizontal gene transfer comprising capsule biosynthesis clusters explains rapid shifts in the expression of surface antigens enabling vaccine escape mechanisms [11,12]. Despite phylogenetic differences, bacteria have also converged on similar complement evasion strategies, including the expression of surface proteins that bind the complement regulator factor H, produce complement inhibitors, or make outer membrane modifications that make the membrane resistant to the membrane attack complex [13,14,15,16]. Antigenic and phase variation generate variant surface repertoires that avoid antibody recognition and permit persistence of the bacterial infection.

## 2. Immune Delay During Infection

Immune delay refers to the ability of pathogens to avoid the induction of an innate host immune response for a number of days, thereby allowing the pathogens to result in a high pathogen load and to induce pathogen defense strategies such as substantial numbers of viruses bound to monocytes resulting in pathogen spread throughout the host, generation of bacterial biofilms, the induction of intracellular bacterial communities, or the induction of modifications of epithelial cell walls protecting against neutrophil recruitment to the infection site. Our hypothesis is that pathogen selection can then optimize the period of delay towards maximum infectivity (and not severity), balancing the height of the pathogen load vs. host, maintaining, on average, social connectivity with potential new hosts. We have summarized in Box 1 the relevant concepts that are discussed in this paper.

Box 1 **Relevant concepts relating to the modulatory activity of LF-EMF exposure to neutrophils and their protective immune activity in infections.**

  Immune delay: Immune delay describes the ability of pathogens to temporarily slow the host’s immune response. Evolutionarily successful pathogens can postpone effective innate and adaptive immunity by evading detection, suppressing key signaling pathways, or diverting immune responses in unproductive directions. Mechanisms such as altered antigen presentation, reduced pattern recognition signaling, or skewed cytokine production allow pathogens to buy time—enabling replication, spread, and persistence before immune control is fully established [1,17].

  Immune evasion: Immune evasion encompasses the diverse strategies that pathogens have evolved to avoid, suppress, or manipulate host immune defenses. These strategies include antigenic variation, inhibition of innate immune sensing, interference with antigen presentation, and resistance to immune-mediated killing. Through immune evasion, pathogens can survive and replicate despite an otherwise functional and competent immune system [18,19].

  Difference between immune delay and suppression of early innate immune responses: Whereas suppression of early innate immunity reflects direct inhibition of immune signaling pathways, immune delay describes a temporally orchestrated postponement of effective immune activation that allows pathogens to gain an early replicative or spatial advantage despite the eventual emergence of robust immune responses. Immune delay refers to a time-based advantage gained by pathogens through transient interference with immune activation because of postponement, not permanent inhibition of the immune responses. Immune delay can occur without complete suppression of innate signaling because of misdirection, dampening, or desynchronization of immune pathways. As a consequence, the pathogen has already gained a foothold [20,21,22,23].

  NETosis: NETosis is an innate immune defense mechanism in which activated neutrophils respond to bacterial or viral pathogens by releasing decondensed chromatin coated with antimicrobial proteins. These neutrophil extracellular traps (NETs) immobilize and help neutralize invading microbes. However, NETosis can be a double-edged sword, as some pathogens exploit or dysregulate this process to enhance immune evasion, drive excessive inflammation, and contribute to tissue damage [24,25].

  Cellular immune responses upon exposure to LF-EMF: The interaction between pathogen exposure and low-frequency electromagnetic field (LF-EMF)-modulated immunity refers to the subtle influence of LF-EMFs on immune cell function during infection. While pathogens activate innate and adaptive immune cells such as neutrophils, macrophages, and lymphocytes, concurrent LF-EMF exposure may modulate intracellular processes including calcium signaling, redox balance, membrane receptor activity, and transcriptional pathways such as NF-κB. These effects can alter the strength, timing, or polarization of immune responses—impacting cytokine release, phagocytosis, and NETosis—without LF-EMFs acting as a direct immune stimulus [26,27,28].

  Type I interferon as a target in neutrophil LF-EMF exposure: In this context, pathogen-derived signals stimulate neutrophils to produce or respond to type I interferons (IFN-α/β), while simultaneous LF-EMF exposure subtly reshapes intracellular signaling networks. Modulation of calcium dynamics, redox status, and transcriptional regulators such as IRFs and NF-κB can influence the magnitude and timing of neutrophil interferon responses. As a result, downstream processes—including interferon-stimulated gene expression, communication with other immune cells, and overall immune polarization—may be fine-tuned during antiviral and antibacterial responses [29,30,31,32].

  Regulation of early immune responses to infection and modulation by exposure to magnetic fields: Neutrophil responses to infection are organized in time by oscillatory calcium and redox signaling that regulate NETosis and may intersect with type I IFN-dependent transcriptional programs through feedback loops. Experimental evidence indicates that low-frequency magnetic and electromagnetic fields can modulate these oscillatory inputs and thereby plausibly altering the timing and magnitude of neutrophil NETosis and IFN-driven responses [27,32,33,34]. Further studies are necessary to validate these concepts.

Innate phagocytes (cells with macrophage- and neutrophil-like functions) and their core effector mechanisms are conserved across animals, whereas classical adaptive systems arose with vertebrates [35]. Because innate immunity acts immediately while adaptive responses require days for clonal expansion, pathogens that affect conserved innate responses gain time to replicate, disseminate, and reduce antigen presentation for adaptive priming. Viral and microbial strategies were developed in evolution that impair interferon signaling, pattern sensing, complement, and programmed cell death pathways [36]. Pathogens can delay, induce, or subvert neutrophil and infected cell death programs to alter early containment, and microbial modulation of neutrophil apoptosis is a known evasion tactic [37].

The induction of innate defenses will limit the infection consequences in the first hours, whereas adaptive responses (antibody formation and T-cell clonal responses) develop later in time. Thus, disabling innate signaling prolongs this early window and can thereby blunt adaptive immune activation [21]. By targeting conserved innate sensors (the release of interferons and complement factors) or effector cells (by the induction of apoptosis), pathogens can delay their detection and reduce efficient priming of adaptive immunity and thereby enhance viral transmission and pathogenesis [2,3]. The period of delay between the initial exposure to a pathogen and the activation of an adaptive immune response leaves the host susceptible to the rapid multiplication and spreading of pathogens, especially in the early stages of infection. Trained or innate immune memory (because of epigenetic or metabolic reprogramming of myeloid progenitors and other innate cells) can shorten this effective delay and improve the early control, although some pathogens have evolved mechanisms to counter this innate memory induction [38]. Manipulation of neutrophil apoptosis or macrophage activation can directly alter early containment and the subsequent inflammatory signals required to recruit and instruct the formation of adaptive immune responses [36].

Several viral pathogens (HIV, LCMV, SARS-CoV-2, influenza A) are able to delay immune activation (to 3–4 days post infection), thereby rendering both innate and acquired immune responses much less effective in fighting infection, as demonstrated dramatically in SARS-CoV-2 infection [39,40,41]. Also, in virulent bacterial infections there is a substantial lag period between the infection and the generation of a pathogen-specific T-cell response, underscoring the relevance of an innate immune response in restricting the uncontrolled growth of the bacteria in the initial phase of infection [42,43,44]. Examples of bacteria that actively delay the induction of an innate immune response are *S. aureus* [45], uropathogenic *E. coli* [46], *Chlamydiae* [47], *Yersinia* species [48], *Listeria monocytogenes* [49], *Streptococcus agalactiae*, *Bacillus anthracis*, *Streptococcus equi*, *Salmonella* AvrA, and *Helicobacter pylori* [50].

These bacterial and viral pathogens deliberately induce an immune delay during the early phases of infection, thereby creating a window of opportunity for the replication and dissemination of the pathogens before robust host immune responses are activated. Several mechanisms have been described: suppression of initial innate sensing by interfering with pattern recognition receptor (PRR) signaling by *Yersinia* species, blocking of phagocytic uptake, and inhibition of TLR-induced NF-κB (nuclear factor kappa-light-chain-enhancer of activated B cells) activation. *Salmonella* and *Shigella* delay inflammatory responses by remodeling lipopolysaccharide structures or injecting effector proteins that dampen mitogen-activated protein kinase (MAPK) and NF-κB pathways [51,52,53]. *Staphylococcus aureus* and Streptococcus pneumoniae secrete molecules that impair complement activation and neutrophil recruitment [54,55]. Viruses employ strategies that postpone host antiviral responses by suppressing the induction of type I interferon (IFN) gene activation and subsequent downstream signaling. influenza A virus NS1, paramyxovirus V proteins, and coronaviral NSP1/ORF6 are well-characterized IFN antagonists that inhibit RIG-I/MDA5 activation, IRF3 phosphorylation, or nuclear translocation of STAT1/STAT2 complexes [56,57,58]. These early blocks reduce antiviral gene expression, delay recruitment and activation of innate immune cells such as neutrophils and monocytes, and allow pathogens to establish higher initial loads that shape downstream immunopathology [29]. However, these activities leave the slower alternative pathways for type I IFN induction intact, thereby inducing activation, albeit delayed, of neutrophils and subsequently the complete immune system [59]. Collectively, these mechanisms highlight that early-phase immune suppression is a conserved and strategically critical component of microbial immune evasion.

## 3. Neutrophils in the Innate Immune Response

Neutrophils are rapid responders, agile and sensitive to external cues, that are mobilized from the bone marrow by chemokine signals. They migrate to infected tissues, where they execute bacterial killing while producing chemokines and cytokines which recruit and instruct other innate and adaptive cells (Box 2).

Box 2 **Summary of the life of a neutrophil [60].**

**Figure 1:**
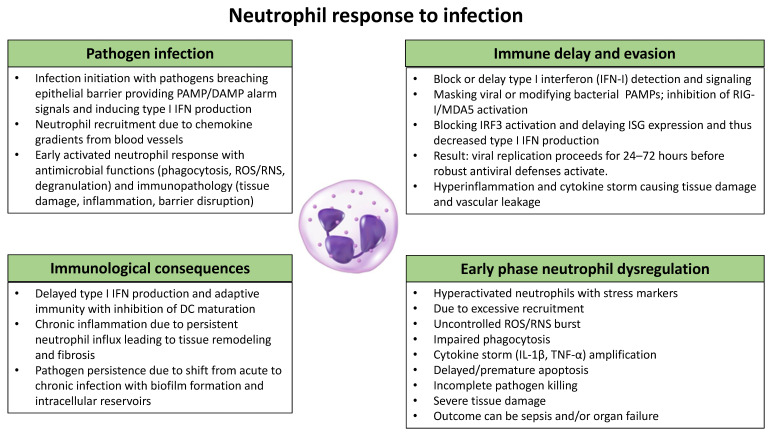
Overview of the role of neutrophils in viral and bacterial pathogen infection, the induction of immune delay and pathogen evasion, the immunological consequences for the host defense, and relevance of early phase neutrophil dysregulation. These mechanisms and characteristics are discussed above.

  The current view is that mature neutrophils in circulation are short-lived with a life span of 7 to 8 days in humans [61] and are replenished at a rate of 1.1 million per second. Mature neutrophils are held, ready to be called upon during infection, in a circulating pool, a marginated pool adhering to vascular walls, and a pool in the bone marrow, containing 20 times the number in circulation [61,62]. In the absence of infection, neutrophils age in a few days and a part of them migrates back into the bone marrow, while another part becomes apoptotic and migrates to liver or spleen, all to be phagocytosed by macrophages. During infection, neutrophils are recruited to the infection site, are primed after recognizing PAMPs, and start producing H_2_O_2_ (which makes them a target for phagocytosis by macrophages). Having trafficked into tissue, they are activated and start degranulation, releasing cytokines and antimicrobial factors stored, but not produced, in vesicles inside the neutrophil. With that, and by NETosis and phagocytosis, they fight the infection, but also cause collateral tissue damage, giving rise to DAMPs. During the fight, they die or become apoptotic and are cleared by macrophages. During infection, the pools are called upon to release additional neutrophils, in severe infections even to the extent of total depletion of mature neutrophils, neutropenia, after which immature neutrophils are emergency released by the bone marrow, which are less controlled and responsible for serious tissue damage, as in the lungs in COVID-19 (Figure 1).

### 3.1. Neutrophil Migration

Neutrophil trafficking is determined by the ratio of CXCR4 and CXCL12 chemokines and adhesion molecules that result in bone marrow retention or mobilization signals. The induction of CXCR4 or the downregulation of CXCL12 is central to mobilization, while CXCR2 ligands and G-CSF drive the release during infection or stress [63]. Once in the vasculature, neutrophils are guided to infected tissue by sequential adhesion, diapedesis, and interstitial chemotaxis, while local endothelial signals profoundly shape neutrophil arrest and retention [64]. In addition, neutrophils can reverse-migrate from tissues and be cleared in marrow or other organs by macrophages as senescent or reverse-migrated cells home for clearance depending on their CXCR4/CXCL12/IL-8 signaling [65].

Neutrophils display robust positive feedback amplification processes that support steering the effective migration towards chemotactic gradients, induce multiple waves of neutrophil recruitment to the site of inflammation and infection, and promote the different effector functions of other cells to provide an integrated antimicrobial immunity [66]. When only a few neutrophils engage and recognize such a pathogen, they will start a cascade of activation of neutrophils (as they are so abundant with about 60% of peripheral blood leukocytes) and other cells, like monocytes, that together provide an efficient innate immune response [66,67]. Neutrophils alter transcriptional and proteomic programs as they transit from marrow to blood and subsequently to tissues, changing the activation of various chemotaxis, survival, and effector pathways that ultimately influence their trafficking and lifespan. Collectively, this explains how infection redistributes neutrophils and the induction of tissue accumulation that can alter peripheral counts and function [68,69,70]. Circulating absolute neutrophil count (ANC) at any moment thereby reflects (a) the rate of tissue extravasation or margination, (b) the rate and magnitude of marrow release (including emergency granulopoiesis), and (c) clearance or sequestration in organs such as lung and spleen. If tissue egress outpaces marrow compensation, a transient fall in peripheral ANC is plausible, but there is not a consistent transient neutropenia across infections [71,72].

### 3.2. Infection Recognition

Neutrophils recognize invading bacteria and viruses by germline-encoded pattern recognition receptors (PRRs) that detect conserved microbial structures and infection-associated signals. Cell-surface Toll-like receptors (TLRs) such as TLR2, TLR4, and TLR5 sense bacterial lipoproteins, lipopolysaccharide (LPS), and flagellin, respectively, while endosomal TLR7/8 and TLR9 detect viral RNA and unmethylated CpG DNA [73,74]. In the cytosol, neutrophils express RIG-I-like receptors (RIG-I, MDA5) that recognize viral RNA and NOD-like receptors (e.g., NOD1, NOD2, NLRP3) that detect bacterial peptidoglycan fragments and cellular stress, leading to inflammasome activation and IL-1β production [75,76,77]. Neutrophils also integrate opsonization and phagocytic signals as Fc receptors bind antibody-coated microbes, while complement receptors (CR1, CR3) mediate recognition and uptake of C3b- and iC3b-opsonized pathogens [78,79]. Additional surveillance pathways include C-type lectin receptors such as Dectin-1 and Dectin-2, which recognize fungal β-glucans but also contribute to sensing complex microbial communities [80]. Neutrophils become activated by two signals: a priming step followed by an activation step. It was proposed that simultaneous ligation of different (at least two) receptors is required for proper activation of neutrophils [81]. The engagement of PRRs triggers the formation of rapid effector responses, including phagocytosis, degranulation, production of reactive oxygen species (ROS), and the formation of neutrophil extracellular traps (NETs), and all of these are essential for early control of both bacterial and viral infections [27,82]. Together, these recognition mechanisms enable neutrophils to function as versatile sentinels that translate microbial detection into immediate antimicrobial action. This suggests that early activation of neutrophils, e.g., by LF-EMF, is a possible target for immune delay mitigation.

### 3.3. Neutrophil Activation and Effector Functions

Upon arrival, neutrophils activate rapid, cell-intrinsic antimicrobial mechanisms that contain microbes before adaptive immune responses are triggered. These effector programs combine phagocytosis and killing via granule fusion and lysosomal degradation; proteolytic and degranulation responses that release proteases and antimicrobial peptides; oxidative burst and ROS generation that stimulate intracellular killing and help signal to neighboring cells; and extracellular trap (NET) formation that immobilizes and slows microbes extracellularly [83,84,85,86,87]. The metabolic support response, based on glucose-driven glycolysis and pentose phosphate pathways pointing to a crucial role of mitochondria, enables these rapid effector functions under infection-driven metabolic reprogramming [88]. Neutrophils act as both effectors and communicators, as their secreted mediators and cell–cell interactions amplify the recruitment of antigen-presenting cells leading to enhanced presentation of antigens to T cells for the induction of later adaptive immune responses [89]. Neutrophil numbers and their lifespan and function are dynamically regulated depending on the phase of the infection, and different neutrophil subsets and clearance pathways ultimately determine whether early responses resolve the infection or drive infection-related pathology. Maintaining homeostasis and effective resolution of the infection rely on neutrophil recirculation, programmed senescence, and macrophage-mediated clearance of dead cells [90]. Activated neutrophils induce the infection-associated inflammatory response by secreting chemokines that promote the further infiltration of additional innate immune cells like macrophages and even more neutrophils. Because neutrophils are attracted in much larger numbers than other immune cells, they have a substantial influence on the outcome of the infection even in tissues where other cells (e.g., macrophages and dendritic cells) are initially more prominently present and productive per individual cell [91].

### 3.4. Death of Neutrophils

Neutrophils do not have a single, fixed trigger for inducing cell death, like a certain number of bacteria that are phagocytosed. Instead, depending on the pathogen-specific activation signal, and the ensuing antimicrobial response, neutrophils undergo a different death program [92]. Even when neutrophils phagocytose only a small number of microbes, they can already proceed to apoptosis, and this ensures that ultimately phagocytosing neutrophils are cleared by macrophages, thereby promoting anti-inflammatory clearance. When phagocytosing multiple bacteria, there is a faster induction of the apoptosis program, which is beneficial for the host as it prevents the release of damaging neutrophil enzymes into the local tissue [93,94]. When activating inflammasome formation, neutrophils can also die by so-called pyroptosis [95]. Neutrophils are able to execute NETosis in two different forms: a slow induction of NETosis over several hours that is associated with decondensation of chromatin and the disintegration of the cellular membrane. This refers to suicidal NETosis which is distinct from vital NETosis. During this NETosis program, NETs are released within minutes and this does not result in the death of the neutrophil and allows the neutrophil to continue moving and phagocytosing particles [96,97].

NETosis can be performed by only a subset of neutrophils in response to strong signals (including IL-8, PMA, LPS, fungi, certain bacteria), after interacting with large extracellular pathogen particles, or when phagocytosis is difficult or appears insufficient. Importantly, NETosis provides an alternative pathway leading to cell death but it also was found not to be required for neutrophil death during infection [98,99].

### 3.5. Connecting Innate to Adaptive Immune Responses

Neutrophils coordinate the crosstalk between the innate and the induction of the adaptive immune response during infection. Upon encountering pathogens, neutrophils release cytokines and chemokines such as IL-12, IL-18, TNF, CCL2, and CXCL10 that shape dendritic cell (DC) maturation, promote inflammatory monocyte recruitment, and influence the quality of T-cell priming in draining lymph nodes [30,89,100,101]. Neutrophils also migrate to lymphoid organs, where they interact directly with DCs, B cells, and T cells. Here they can also deliver microbial antigens to DCs or even acquire antigen-presenting capabilities by expressing MHC class II and co-stimulatory molecules that enable limited T-cell activation [102,103,104,105]. In addition, neutrophil-derived NETs and granule proteins enhance antigen availability and adjuvanticity, promoting antibody responses, while B-cell-helping neutrophils in the splenic marginal zone can drive class-switching and plasmablast differentiation during bacterial infection [106,107,108]. Through these diverse mechanisms, which include modulating APC (antigen-presenting cell) function, shaping cytokine milieus, trafficking into lymphoid tissues, and influencing antibody production, the neutrophils act as essential intermediaries that translate early innate sensing into effective and tailored adaptive immunity. As such, the activation state and speed of response of neutrophils has a profound influence on reducing incubation time and the delay of the immune response to a new infection, and thereby on the maximum size and extent (and damage caused) that the infection can attain.

## 4. Neutrophils in Infection-Induced Evasion and Immune Delay

### 4.1. Virus-Induced Type I Interferon Response

The secretion of type I interferons (IFNs) is characteristic of the start of an antiviral immune response to a virus infection, and these IFNs do not act directly on the virus, but induce gene activation and the secretion of proteins that inhibit the replication of the virus and also increase the numbers of activated and virus-cytotoxic CD8^+^ T cells [109]. Type I IFNs are a family of cytokines with one subtype of IFN-β, 13 subtypes of IFN-α, and in addition IFN-ω, IFN-κ, IFN-ε, and IFN-ν. Type III IFNs (IFN-λ) are released rapidly after the start of the infection, sharing many functions. A viral infection will be detected by PRRs (RIG-I, MDA5, TLRs) resulting in the activation of transcription factors (including IRF3, IRF7, NF-κB), and eventually resulting in interferon-mediated gene transcription and protein production associated with interferon secretion and autocrine and paracrine signaling. As a consequence, the JAK-STAT pathway will be activated and the expression of interferon-stimulated genes (ISGs) will be upregulated, leading to the establishment of the characteristic antiviral state. Unlike type I interferons, type III interferons primarily act on epithelial surfaces, making them particularly relevant for respiratory viral infections [110]. At the mucosal surfaces in the airways and the gastrointestinal tract, this early innate antiviral IFN response can block virus replication [111,112,113]. The existence of multiple IFN-α genes and the fact that virtually all viruses eventually encode proteins that antagonize IFN production or their response to type I IFNs emphasizes the importance of IFN during the induction of an antiviral immune response. Virus-encoded proteins can cause a delay of type I IFN secretion until a few days after infection, coinciding with the end of the incubation period [114]. Activated neutrophils rapidly produce type I IFNs [115] that prime the innate immune system to maintain a preactivated antiviral and antibacterial state which is further supported by the large secretion of type I IFNs by tissue resident plasmacytoid dendritic cells [29,116,117]. In addition, these dendritic cells induce the production of type II interferons (IFN-γ) by natural killer (NK) cells, T cells, and macrophages. These interferons thereby connect the innate and the adaptive antiviral immune response, resulting in the induction of apoptosis of virus-infected cells while at the same time activating macrophages, NK cells, and T cells.

Activated neutrophils, besides macrophages and DCs, can assemble the NLR family member of pyrin domain-containing 3 (NLRP3) inflammasomes in response to microbial infection and cellular damage, thereby mediating caspase-1 activation and the secretion of pro-inflammatory cytokines, including IL-1β/IL-18 [118,119]. However, the increased production and their type I and II IFN-induced target gene expression, in addition to the aberrant activation of the NLRP3 inflammasome, can result in disorders associated with severe inflammation, but also in the induction of a stronger and faster early immune response against infections resulting in the increased presence of preactivated neutrophils. This increased resistance against, e.g., SARS-CoV-2 thereby mitigates the development of severe COVID-19 disease [120,121,122]. An early interferon response, within 24–48 h, generally correlated with mild disease, while delayed responses (>5 days) were associated with severe COVID-19 disease and a higher mortality. This difference in kinetics provides a narrow therapeutic window for interferon treatment between 0–3 days after the onset of symptoms [115].

Different viruses have evolved mechanisms to evade and suppress the crucial interferon responses, contributing to the observed delays in the induction of antiviral immunity. The low interferon response is the consequence of viral proteins that suppress this response in neutrophils and dendritic cells, push macrophage polarization towards the anti-inflammatory type 2 phenotypes, and impair natural killer cell activation and function. Multiple viral structural and non-structural proteins have been identified to act as interferon antagonists [123] or by inhibiting viral RNA detection by RIG-I, MDA5, and TLR3 and TLR7 signaling in endosomal compartments. The inhibition of IRF3 and IRF7 nuclear translocation and activation and suppression of NF-κB signaling pathways interfere with the transcriptional machinery that is required for interferon gene expression. In addition, the suppression of interferon responses can occur after transcription by mRNA degradation and inhibition of translation and interference with JAK-STAT signaling cascades resulting in the suppression of interferon-stimulated gene expression. In SARS-CoV-2 infection, patients with mild to moderate COVID-19 symptoms showed a rapid induction of interferon shortly after the onset of symptoms with peak interferon levels and a coordinated upregulation of interferon-stimulated genes (ISGs) occurring during the first week of illness [112,124]. In contrast, infected patients who develop severe COVID-19 disease showed a characteristic pattern of a delayed and dysregulated interferon response, leading to suppressed interferon responses during early phases of the infection. The dysregulated and late-onset interferon signaling was associated with tissue damage and an impaired resolution of interferon responses during the recovery phase. Severe COVID-19 cases displayed lower IFN-α levels with reduced ISG expression despite high viral loads, with associated delayed peak interferon responses [124].

Low viral loads permit the induction of effective interferon responses and a subsequent virus control, while delayed interferon responses cause high viral loads that increase even further due to pathological feedback loops [124]. Delayed interferon induction will thus allow further viral replication resulting in higher viral loads that even further enhance interferon suppressive mechanisms and contribute to increased tissue damage rather than viral control [125,126,127]. Eventually, the viral infection can disseminate systemically and overwhelm local immune responses.

The induction of pre-activated neutrophils and innate immune activity might suppress the early exponential viral replication phase and thereby keep the peak viral load lower and preserve the synchrony between the induction of subsequent innate and adaptive immune responses. One possibility to induce pre-activated neutrophils could be LF-EMF such as in [128,129]. In addition, such treatment alters the early viral growth and the host response timing, thereby shrinking the window for uncontrolled exponential replication. Pre-activation interrupts the positive feedback where high early viral replication delays innate sensing, allowing the virus to outcompete host responses. However, inducing high interferon levels before the actual infection may induce possible negative effects, including the inhibition of cell proliferation and induction of tissue damage, thereby arguing for controlled, localized, or transient priming strategies early during infection rather than prolonged systemic IFN elevation [125,130].

### 4.2. Bacterial Induced Type I Interferon Response

Similar to viral immune defense, a preactivated antibacterial state can be expected to mitigate diseases, like UTI largely caused by uropathogenic Escherichia coli (UPEC). Upon infection, the bladder-tissue-resident macrophages secrete chemokines to recruit neutrophils in very large numbers, and both types of sentinel cells also secrete TNF-α, allowing the neutrophils to cross the epithelial barrier. Macrophages also activate the local microenvironment resulting in inflammation by the locally generated TNF-α. Therefore, the immune response of the urinary tract needs to balance a protective immunity against microbial challenges with control of the inflammatory response to maintain the structural integrity of the epithelial barrier. This balance can result in premature termination of inflammatory responses, leading to the persistence of residual bacteria, and can potentially give rise to chronic or recurrent infections, which are remarkably common in the urinary tract [131].

Neutrophil phagocytosis exhibits a biphasic kinetics. First, there is a receptor diffusion-limited phase that is characterized by the slow initial engulfment of the captured pathogens. This rate-limiting step involves receptor–ligand interactions and membrane reorganization processes and continues until completion of approximately 50% engulfment. The second phase is a much faster signaling-driven rapid phase with an acceleration of the engulfment process that is mediated by active receptor recruitment and activation of intracellular signaling cascades [132,133]. Neutrophil-mediated clearance of engulfed bacteria occurs within the first 8 h of infection while NET formation occurs within minutes to hours (30 min–8 h) depending on the bacterial stimulus. Typically, 60–80% of neutrophils form NETs under optimal conditions within 2–4 h. However, different bacterial species elicit varying NET formation kinetics and intensity.

Neutrophil responses for antibacterial immunity can be delayed or misdirected by pathogen-driven modulation of their recruitment, survival, and interactions with other immune cells, and these altered behaviors create temporal windows that permit bacterial survival and dissemination. Several pathogen strategies and host signals cause neutrophils to become modulators for the induction of protective T-helper-cell-mediated adaptive responses [89]. Biofilm-producing bacteria impair chemotaxis of neutrophils, thereby delaying neutrophil invasion [134]. Alternatively, some bacteria induce the expression of anti-inflammatory cytokines (including IL-10) that inhibit neutrophil recruitment and bacterial killing, in addition to initiating the reduction of ROS production, and together resulting in prolonged bacterial persistence in local tissues [135]. Stimulation of the IL-10/IL-10 receptor axis restrains neutrophil recruitment and killing (by the production of ROS and serine proteases) and thereby prolongs bacterial persistence. Some bacterial strains induce activation of neutrophil death (apoptosis, NETosis, necrosis). Other strains delay neutrophil death resulting in prolonged presence of neutrophils or the release of immunomodulatory remnants (as the result of lysed neutrophils or material released by NETosis) that impede the generation of a coordinated immune response [136]. For example, *Staphylococcus aureus* can kill or inhibit neutrophil function by producing pore-forming cytolysins that create membrane pores and phenol-soluble modulins that lyse neutrophils and escape neutrophil extracellular traps (NETs) by secreting a chemotaxis-inhibitory protein that prevents activation, as well as secreting leukocidins that specifically target immune cells [137]. Streptococci employ neutrophil resistance strategies that evade neutrophil killing, including the release of capsular polysaccharides that resist phagocytosis, other surface proteins that prevent neutrophil adherence, and secreted proteases and toxins that degrade neutrophil-released antimicrobial factors as well as a zinc metalloproteinase that prevents neutrophil extravasation [137].

In addition, specific bacterial factors can alter mitochondrial depolarization and caspase activation to regulate phagocytosis and reduce bacterial opsonization, thereby inhibiting effective recognition by complement receptors and Fc receptors on human neutrophils [137]. Specific bacterial infections, including Acinetobacter baummanii, display the ability to adhere to neutrophils without being taken up and subsequently killed by phagocytosis. From the infection site, neutrophils can transmigrate back to the vasculature, and this reverse migration of neutrophils actually helps the bacteria to disseminate the infection to other organs [138]. Protective immunity to these bacteria is primarily mediated by specific antibody formation interacting with Fcγ receptors, thereby allowing enhanced opsonization and phagocytosis of these bacteria by innate immune cells, including neutrophils themselves [139].

## 5. Dysregulated Neutrophils in Infectious Diseases

### 5.1. Dysregulation Resulting in Immune Evasion

Viral and bacterial immune evasion strategies profoundly modulate both neutrophil number and function. Many pathogens possess the ability to manipulate the recruitment of neutrophils by altering chemokine networks or, alternatively, by interfering with endothelial adhesion due to the use of complement. These mechanisms limit the influx of neutrophils to sites of infection or, conversely, drive excessive and tissue-damaging accumulation. Once recruited, the lifespan of neutrophils is actively altered as a range of viral infections (e.g., HIV, influenza, RSV) and bacterial products (e.g., flagellin) can either delay or, alternatively, accelerate neutrophil apoptosis, thereby extending survival to sustain an inflammatory niche or prematurely removing these cells to blunt bactericidal capacity [140,141,142]. In parallel, pathogens produce virulence factors that impair neutrophil effector functions, including chemotaxis, phagocytosis, degranulation, and oxidative burst, or that reprogram neutrophils toward immunoregulatory phenotypes with reduced antimicrobial activity. A prominent example is the targeting of neutrophil extracellular traps (NETs): various bacteria and viruses express nucleases, surface capsules, or NET-binding proteins that either prevent NET formation and promote their degradation, or neutralize the NET-associated activities of histone proteins and proteases, and thereby escaping extracellular killing [143,144].

*Staphylococcus aureus* leukocidins, *Yersinia* YopH, and other secreted effector molecules can directly inhibit the recruitment of neutrophils, activation of their signaling pathways, and bactericidal functions to promote neutrophil survival in tissues and in biofilms [145,146]. In respiratory viral infections, including influenza and SARS-CoV-2, viruses alter the magnitude and quality of neutrophil responses, thereby skewing them either toward a hyperinflammatory state or, alternatively, to a dysfunctional state, and this contributes to enhanced pathology while failing to clear the infection efficiently [147,148,149,150]. Collectively, these strategies underscore that modulation of neutrophil production, trafficking, lifespan, and effector functions is central to microbial immune evasion strategies which largely determine the outcome of the infection [151,152].

### 5.2. Aberrant Neutrophil-Based Innate Immune Responses

Pathogen-induced infections generally cause rapid and massive production of various pro-inflammatory cytokines, such as TNF-α, IL-1β, IL-6, IL-12, IFN-α, IFN-β, IFN-γ, monocyte chemoattractant protein-1 (MCP-1), and IL-8. However, these cytokines need to be balanced with anti-inflammatory cytokines, like IL-10 and TGF-β, in order to prevent the formation of a harmful inflammation. In an overactive immune system, acute systemic infection can induce a cytokine storm in which these pro-inflammatory cytokines are strongly elevated, causing systemic inflammation (associated with fever) and multiorgan dysfunctions, and even systemic organ failure and death [153]. Dysregulation of neutrophils leads to delayed induction of apoptosis and results in the accumulation of these neutrophils in the infected tissues, the increased release of their cytotoxic products, the development of local tissue damage, and the induction of inflammation [154].

Evidence increasingly supports a role for neutrophils in the development of a life-threatening severe inflammation in the lungs, leading to fluid accumulation and impaired oxygen exchange, called ARDS (acute respiratory distress syndrome) and also vascular thrombosis, which occur in patients with severe COVID-19. Prolonged neutrophil activation, associated with the induction of a delayed apoptosis and increased neutrophil extracellular trap (NET) formation, are all linked to alveolar damage and adverse outcomes. Immunophenotyping of peripheral blood from COVID-19 patients revealed a significant left shift in the ratio between immature and mature neutrophils associated with disease severity, indicating the presence of immature neutrophils pointing towards severe inflammation [155]. The increased numbers of immature neutrophils in patients with severe COVID-19 and the disappearance of mature neutrophils likely reflect gradual and sustained mobilization of these cells into the lungs (with granulocytes comprising up to 80% of cells), leading to premature release of immature neutrophils from the bone marrow. This increase strongly correlates with disease severity and is associated with elevated IL-6 and IP-10 levels, two key players in the cytokine storm [156,157,158,159].

In summary, an inadequate early immune response enhances pathogen immune evasion, virus and bacterial propagation, the spread of infection and subsequently local cell death, and the release of PAMPs and DAMPs. As a consequence, these signals can trigger a later uncontrolled local and even systemic inflammation and substantial tissue damage and subsequent disease as in SARS-CoV-2 infection [160]. The aberrant immune responses underlying severe COVID-19 disease were found to be based on early and persistent activation of neutrophils associated with impaired type I IFN (IFN-I) signaling in circulating cells, illustrating the need for a controlled downregulation of neutrophil activation after severe COVID-19 develops and patients are hospitalized [161] (Figure 1). Multiple infectious diseases, including bacterial pneumonia, influenza, tuberculosis, herpes zoster, and most recently COVID-19, are all more prevalent among the elderly, leading to increased morbidity and mortality. This is due to aging, with infection history causing cell signaling between immune cells to be impaired and the initial immune response becoming weakened [162,163,164].

## 6. Electromagnetic Fields (EMFs) and Neutrophil Activation

### 6.1. Biological Effects of LF-EMF Exposure

A substantial body of in vitro evidence indicates that even short-term modest exposure to low-frequency electromagnetic fields (LF-EMFs) can modulate cellular signaling pathways. See Box 3 below. Refs. [165,166] demonstrated that mammalian cells respond to field strengths as low as ~0.15 μT, highlighting the sensitivity of intracellular signaling machinery to exposure to such weak, non-thermal stimuli. Across several studies, NADPH oxidase (NOX2) appears to be the most consistent response candidate allowing the analysis of LF-EMF-induced cellular responses, particularly because of its role in regulating the production of reactive oxygen species (ROS) [128,165]. In neutrophils, LF-EMF stimulation results in increased ROS production within minutes after exposure. Poniedziałek et al. [167] observed a detectable increase in enhancement of oxidative burst after only 15 min of LF-EMF exposure, indicating the rapid engagement of NOX-dependent pathways. Importantly, LF-EMF exposure can induce activation of MAPK signaling leading to upregulation of ERK1/2 (extracellular signal-regulated kinases), and this occurs without promoting excessive cellular proliferation, and is therefore considered not to be oncogenic [165]. LF-EMF exposure enhances neutrophil extracellular trap (NET) formation ex vivo. This effect appears to be ROS-dependent and mediated through the NADPH oxidase pathway. Notably, enhanced NETosis was only observed when LF-EMF exposure occurred in the presence of a strong co-stimulatory trigger, e.g., exposure to the protein kinase C activator phorbol 12-myristate 13-acetate (PMA), and this suggests that LF-EMFs can amplify, rather than independently initiate, the activation of neutrophils [128]. The presence of a pathogen can represent such a biologically plausible trigger that could synergize with LF-EMF exposure during the induction of an early innate immune responses to an infection.

Box 3 **Basic aspects of EMF bioeffects.**

  Electromagnetic fields (EMFs) consist of coupled electric and magnetic components. A time-varying magnetic field induces an electric field that can generate microcurrents in conductive biological tissues. EMFs below ~100 kHz and within ICNIRP and WHO safety limits are *non-ionizing* and *non-thermal*: they cannot break chemical bonds, alter molecular structure, or produce physiologically relevant heating [168].

  Despite their low energy, such fields may interact with cells through several biophysically plausible mechanisms. Proposed pathways include modulation of voltage-gated Ca^2+^ channels (VGCCs) [165] and alterations in redox signaling that can influence NOX2-dependent ROS production [128,167,169,170]. Weak magnetic fields can also modulate radical-pair electron spin chemistry in flavoproteins—best established in migratory bird magnetoreception—but this mechanism is still under investigation in mammalian cells [171].

  A growing body of in vitro and in vivo evidence shows that LF-EMFs can modulate immune cell signaling. Reported effects include mild NOX-mediated increases in ROS production [169], enhanced NET formation under co-stimulation (e.g., PMA) [128], transient activation of MAPK pathways (ERK1/2, p38) without driving proliferation [165,172], and altered cytokine secretion such as IL-8 or TNF-α [166,172]. These responses remain within physiological ranges and do not induce DNA damage or cell transformation [165,168].

  Targeted immunomodulation via LF-EMF exposure has been evaluated in in vitro and animal studies that showed promising effects of LF-EMF exposure in conditions such as chronic pain [173], bone injury [174], wound healing [175], and arthritis [176]. Effectiveness of LF-EMF in infection has also been demonstrated in coccidiosis infection in chickens by decreasing the severity of lesions caused by the parasite [177], and in survival studies with infected goldfish [178].

  Collectively, these findings indicate that LF-EMF exposure within safety guidelines can produce subtle but reproducible immunomodulatory effects, including priming of neutrophil-associated pathways relevant for early innate immune activation.

### 6.2. Neutrophils and LF-EMF Exposure

Neutrophils are highly reactive innate immune cells characterized by rapid mobilization, swift signal integration, and pronounced sensitivity to physicochemical cues. These properties make them plausible cellular targets for studying the immunomodulatory capacity of extremely low-frequency electromagnetic fields (ELF-EMFs). Evidence from human studies supports that peripheral blood neutrophils exhibit a measurable activation following a 30 min in vivo exposure to a weak, non-thermal ELF-EMF signal that is delivered at intensities well below WHO and EU safety guidelines. In clinically healthy individuals, this exposure resulted in a rapid and detectable shift in neutrophil activation status, reflected by a decrease in cellular granularity—an established marker of early neutrophil activation [60,129].

Experimental work directly addressing neutrophils as targets of electromagnetic fields (EMF) is still limited, but several in vitro and in vivo studies indicate that both extremely low-frequency (ELF) and high-frequency fields can modulate neutrophil number and effector function in ways that are highly dependent on frequency, waveform, and field strength. Using single-cell microscopy, Rosenspire et al. showed that very weak ultra-low-frequency pulsed magnetic fields (up to 4 G, inducing electric fields ≥ 10^−4^–10^−5^ V/m) phase-locked to the intrinsic ~20 s metabolic oscillation of human neutrophils could either amplify or collapse oscillations in NAD(P)H and flavoprotein redox state, leading to corresponding increases or suppression of ROS and nitric oxide production in adherent, motile cells. These effects required extracellular Ca^2+^, suggesting that induced electric fields couple into Ca^2+^-dependent signaling pathways [179]. In a complementary in vitro study, Poniedziałek et al. exposed human peripheral blood neutrophils to low-frequency EMF (Viofor JPS system; 10–60 μT) and found that such fields modulated oxidative burst as the ROS production was slightly reduced in resting neutrophils, but significantly increased in PMA-stimulated cells, consistent with EMF-dependent “priming” of the respiratory burst rather than direct activation [167].

Neutrophil extracellular trap (NET) formation can also serve as a sensitive read-out of EMF effects. Golbach et al. demonstrated that exposure of human neutrophils ex vivo to a complex low-frequency EMF (four block waves at 320, 730, 880, and 2600 Hz, 300 μT) significantly enhanced PMA-induced NET formation (~25% increase in extracellular DNA at 4 h) and increased superoxide production. In addition, pharmacologic inhibition showed that this effect strictly depended on the NADPH oxidase pathway, implicating EMF-induced augmentation of ROS generation as the proximal driver of enhanced NETosis [128]. These NETs increased killing of *E. coli* but also caused apoptosis of epithelial cells, underscoring that EMF-driven NETosis may shift the balance between antimicrobial defense and collateral tissue damage at infected sites. In contrast, another recent study using a clinically applied 16 Hz extremely low-frequency pulsed EMF (ELF-PEMF) optimized for fracture healing found that, in human neutrophils, this field did not induce ROS or Ca^2+^ influx and did not trigger NET formation by itself; instead, short pre-exposure reduced DNA release in NETs induced by PMA, LPS, or H_2_O_2_ without obvious neutrophil toxicity [180]. Together, these NET-inducing studies suggest that some LF-EMF waveforms can prime neutrophils to exhibit exaggerated NETosis, while other well-defined ELF-PEMF exposure schemes may dampen NET release, thereby potentially limiting subsequent immunopathology during sterile injury or infection.

Importantly, not all neutrophil functions are susceptible to EMF in these models. In neutrophil-like HL-60 and PLB-985 cell lines, Golbach et al. showed that neither a 50 Hz sinusoidal field nor the same Immunent block-wave LF-EMF used in NET studies (5–500 μT) altered the chemotactic Ca^2+^ mobilization, Ca^2+^-related gene expression, and microvilli morphology after short (30 min) or prolonged exposure, arguing against a generalizable effect of LF-EMF on neutrophil Ca^2+^ signaling [169]. At the other end of the spectrum, Vlasova et al. reported that exposure of human whole blood or isolated neutrophils to millimeter-wave extremely high-frequency radiation (32.9–39.6 GHz, 100 W/m^2^ for 15 min) enhanced the oxidant production, morphology, and functional activation in response to opsonized zymosan or *E. coli*, whereas EMF alone had little effect. Using careful controls implicated that subtle sample heating was likely the dominant mechanism [181]. These data highlight that EMF can modulate the magnitude of neutrophil responses to infectious stimuli (e.g., increased oxidative burst and activation markers) but that effects are often dependent on co-stimulation and may be mediated by thermal as well as non-thermal mechanisms.

In vivo findings are consistent with an immunomodulatory role of ELF-EMF on cells, including neutrophils. In a mouse model exposed for one week to a composite ELF-EMF (20–5000 Hz, 10 μT; 1–24 h/day), de Kleijn et al. observed significantly increased circulating leukocyte counts in the group exposed 24 h/day, largely due to elevated neutrophil and CD4^+^ lymphocyte numbers, accompanied by reduced plasma ACTH (adrenocorticotropic hormone) and down-regulated expression of hypothalamic–pituitary–adrenal (HPA) axis genes [182]. These data suggest that whole-body ELF-EMF can indirectly alter neutrophil availability through neuroendocrine mechanisms, potentially affecting early leukocyte recruitment to sites of infection or tissue damage. Conceptually, such EMF-induced shifts in neutrophil numbers and priming thresholds could shorten or prolong the “immune delay” phase during an infection and modulate the balance between pathogen clearance and bystander tissue injury, but this remains largely inferential as direct infection models under controlled EMF exposure are scarce [183].

Overall, the available literature indicates that neutrophils are bona fide EMF targets: specific low-frequency pulsed fields can resonantly modulate metabolic oscillations, ROS, or nitric oxide production and NET formation, while whole-body ELF-EMF exposure alters circulating neutrophil counts via HPA-axis modulation, and high-frequency fields can intensify oxidative responses to particulate microbial agonists through thermal mechanisms [179]. However, the number of well-controlled studies is small, exposure conditions are highly heterogeneous, and most work has been performed ex vivo or in non-infectious settings. For infectious disease contexts, these results should therefore be interpreted as mechanistic proof-of-principle: EMF exposure has the potential to tune neutrophil effector functions (chemiluminescence/oxidative burst, NETosis, availability in the circulation), thereby influencing both antimicrobial immunity and immunopathology, but rigorous in vivo infection models will be required to define dose–response relationships and clinically meaningful effects.

As reported in the literature, variations in signal and environment parameters may have a substantial influence on the effectiveness of immune modulation by an LF-EMF stimulus. The authors have limited themselves to as little variation as possible and used exposures as reported in [129]. In previous studies [129,178], it was reported that a stimulus of 30 min exposure at a 5 μT nominal field strength with fixed frequency contents was effective over a larger range of amplitudes than 0.1 to 50 μT, allowing a non-uniform field produced by flat exposure coils to be used. In addition, application near large amounts of iron and (construction) steel was avoided in order not to introduce large deviations in the North European free-field static ambient field strength of 47 μT.

**Figure 2:**
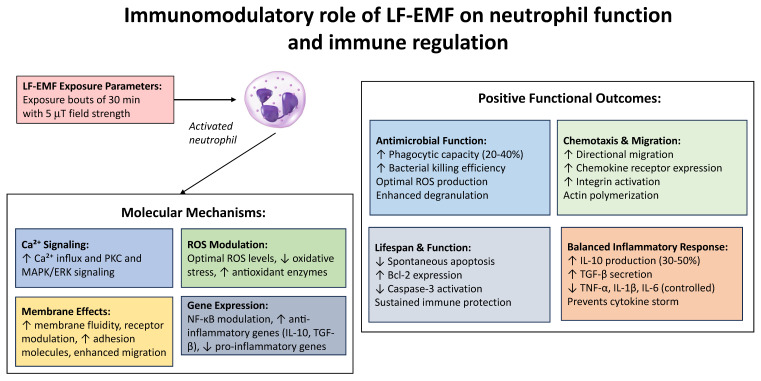
Overview of the immunomodulatory role of LF-EMF exposure on neutrophil function and the consequences for immune regulation as discussed in this review.

Early neutrophil activation induces a priming state in which the exposed cells remain morphologically in a “resting state” yet exhibit enhanced responsiveness to subsequent stimuli. Primed neutrophils display accelerated signal transduction, more efficient pathogen recognition, and faster deployment of effector responses upon encountering subsequent microbial or inflammatory triggers [172,184]. In tissue microenvironments, cytokines and growth factors such as GM-CSF and type I interferons (IFN-α/β) further modulate neutrophil phenotype and function, promoting enhanced ROS generation, degranulation, and directed migration toward chemotactic signals originating from inflamed or infected sites. ELF-EMF-induced priming may therefore act as an early augmenting signal that synergizes with physiological mediators to accelerate the induction of innate immune activation upon pathogen encounter (Figure 2).

## 7. Discussion

The evidence referred to so far supports the thesis that the causative pathogens of several, if not all, large-scale infectious diseases potentially evade the induction of a protective immune response for a limited, but not a large, number of days. That suggests that a limited immune delay is to the advantage of the pathogen and the selection of pathogenicity of infectious organisms is towards the successful induction of such an immune delay, and not complete immune evasion. A host that remains socially active can efficiently infect subsequent hosts, especially when the pathogen load is high and transfer-supporting symptoms arise because of the ensuing immune response. For humans, “socially active” obviously means “going to work or other gathering places”, but for, e.g., migratory birds like cranes with H5N1 avian influenza infections it can mean roosting together in shallow water during the night [185]. The pathogen variant that optimally employs immune delay will become dominant especially when it provides cross-protection to the host against other variants of the same organism.

The induction of a faster and stronger innate immune response, as suggested above, could reduce the infection-related immune delay and sooner and more effectively initiate protective secondary immune responses. Such a treatment can thus be expected to reduce the period that the infection can exponentially grow unhindered and thus keep the infection and its related disease induction much smaller and easier to overcome by the patient’s own immune system [186].

Localized, mild infections are resolved before they induce hematopoietic stress and the granulopoiesis in the bone marrow remains in steady state without a need for the induction of a left shift [187]. On the other hand, a strong and overwhelming, and often systemic, infection utilizes mature neutrophils, thereby inducing neutropenia and emergency granulopoiesis characterized by the release of immature neutrophils into the circulation [188]. As expected, immature neutrophils are less efficient in antimicrobial defense but induce a pro-inflammatory cytokine-secretion profile which is associated with endothelial dysfunction contributing to the development of a more severe disease [149]. Thus, the prolonged presence of immature neutrophils can exacerbate tissue damage and organ failure. Thus, the rapid containment of infection by the induction of an early innate immune response is critical to prevent the exhaustion of the neutrophil pool and also prevents the development of neutropenia and the subsequent release of immature, dysregulated neutrophils, and thereby reduce collateral tissue damage [189]. Thus, keeping the infection small by early immune activation could also prevent neutropenia, the disappearance of mature neutrophils, and the subsequent release of immature, less controlled neutrophils causing more tissue damage. Neutrophil activation critically results in the production of type I IFN which also activates pDCs. Moreover, after the initial IFN response, a crucially important second-wave IFN production occurs, e.g., in epithelial cells [190].

The EMF stimulus as discussed in [129] provides a rapid activation of neutrophils in human volunteers. Therefore, we hypothesize that during an infection, short-term LF-EMF exposure could enhance innate immune responses, modulate cytokine profiles, and reduce the ensuing tissue damage, the development of clinical symptoms, and the need for antibiotics. The defined short-term positive effects of LF-EMF exposure might reduce the risk of inducing a cytokine storm or even triggering the manifestation of autoimmune disease because both are related to severe infection and an ensuing very strong immune response, situations that could possibly be avoided by early immune activation. In summary, severe infections are often poorly controlled and therefore characterized by a delayed or dysregulated immune responses. Therefore, we hypothesize that LF-EMF-exposure-induced early immune activation could contribute to reducing the risk of these complications by limiting pathogen burden and the need for excessive late-stage immune activation.

This suggests that LF-EMF exposure can be applied prophylactically or early during infection to mitigate the consequences of infection-dependent and neutrophil-based immune delay. In addition, activated neutrophils would indeed sooner induce the protective adaptive immune response which would provide complete recovery with less necessity to use supporting antimicrobials. However, the implications of the EMF-induced neutrophil changes for infection outcomes and immunomodulation remain poorly defined. Key mechanistic gaps include whether EMF-stimulated neutrophils change their chemotaxis, phagocytosis, degranulation, or lifespan in infected tissues, and how these functional alterations affect antigen presentation, cytokine milieu, or immune cell crosstalk. For example, although NET formation is enhanced under LF-EMF in vitro, whether that translates into improved microbial clearance or instead exacerbates tissue damage or immunopathology in vivo has yet to be evaluated. Moreover, many of the EMF–neutrophil studies use isolated cells under artificial exposure conditions, and it remains uncertain how clinically relevant exposure parameters (frequency, flux density, duration, waveform) map onto infectious scenarios.

In summary, while there is intriguing evidence that exposure to LF-EMFs can modulate neutrophil function and by extension immune responses during infection, further research is required to clarify the directionality (beneficial vs. harmful), magnitude, and context-dependency of these effects. Proposing neutrophil activation as a therapeutic option in infectious disease raises the question whether this approach may induce neutrophil over-reaction and damage as often reported in severe infections, like severe COVID and influenza A [191]. However, these reports, such as [192], consider the episode to start at hospital admission, when a severe infection is already established. So, when it is stated there that it is imperative to regulate the initially (2 days) increased numbers of neutrophils down to their normal values in blood and tissue by days 4 to 7, they refer to a timing relative to hospitalization, not to the start of infection. Early activation relative to the start infectious disease is meant to prevent a severe infection from being established and is therefore not contradicted by the COVID-19 reports.

## 8. Conclusions

Immune dysregulation in infections generally starts early in the disease course and propagates during the process, and this is generally based on impaired interferon signaling. The advantages of a faster induction of an innate immune response early in a serious infection far outweigh the possible disadvantages because both the maximum infection and the maximum immune response can remain smaller and will be more optimally controlled, and the release of immature neutrophils is reduced or prevented. LF-EMF exposure induces a more rapid innate neutrophil activation with moderate neutrophilia, but the level of neutrophil activation will not be greater than a normal level of neutrophil activation upon infection, with positive effects of controlling infectious spreading and induction of pathology. Thus, activating the innate immune system by LF-EMF, in particular neutrophils, prophylactically or early, could mean fewer infections and more self-cure without antimicrobics, or with less or less advanced antimicrobics, thereby preventing the build-up of antibiotic resistance.

## Figures and Tables

**Table 1:** Annotated table of recurring abbreviations.

Neutrophil	Neutrophillic Granulocyte	Short-lived, free-ranging, and self-propagated phagocytic white blood cells with important functions in the innate immune system. Characterized by their granules containing cytokines and antimicrobial compounds that are excreted when activated. They phagocytose (eat) bacteria- and virus-infected cells and produce NETs, involved in the early phase of inflammation.
NET, NETosis	Neutrophil Extracellular Trap (formation)	NET formation allows neutrophils to kill extracellular pathogens while minimizing damage to host cells by releasing sticky clouds of DNA material and granule-derived enzymes that catch and kill microbes.
PAMPs and DAMPs	Pathogen- and Disease-Associated Molecular Patterns	Cells of the immune system employ PRRs and TLRs to recognize pathogens and damaged host cells and related proteins. PAMPs are derived from microorganisms while DAMPs are originally intracellular proteins or nucleic acids released upon cell death.
PRRs & TLRs	Pattern Recognition Receptors and Toll-Like Receptors	Immune cells express receptor proteins in and on the cell membrane that recognize PAMPs & DAMPs and thereby modify cell function such as activation and cytokine production and/or excretion. Different TLRs recognize different (types) of non-self material and provide rapid innate immune responses.
RIG-I, MDA5, NOD1/2, NLRP3, CD8^+^, RIG-I, MDA5, NOD1/2, NLRP3, Fc/Fcγ receptors, CR1/3, Dectin-1/2		PRRs in and on immune cells that aid in recognizing viral components that initiate the ensuing immune response, while bacterial PAMPs and cellular DAMPs trigger inflammation, and cytokine release (like IL-1β, IL-18) by activating NF-κB/MAPK signaling transduction pathways.
Cytokine		Cytokines are signal proteins with immune regulatory functions and are released by cells and act by binding to specific receptors.
IFN	Interferon	Interferons are a family of cytokines with different types and subclasses important against viral replication and for coordinating immune responses.
CXCxx	C-X-C Motif Chemokine	Chemokines are cytokines with specific functions determining cell trafficking, including neutrophil release from the bone marrow.
Cellular signaling pathways named after/producing NF-κB, MAPK, JAK-STAT, NADPH, MyD88		A process by which chemical or physical signals at the cell surface are transmitted through a cell to the DNA by a complex network of molecular interactions. This result changes in the transcription or translation of genes, and post-translational and conformational changes in proteins that will be secreted affecting their function.
IRF3, IRF7, NF-κB, AP-1, GATA-3, T-bet		Transcription factors are DNA-binding proteins that affect and regulate the transcription rate of genes that are driving the immune response. Different bacteria and viruses can eliminate some of these as an immune delay strategy, thus sabotaging the related pathway.
IL8/10/12/18/1β, TNF	Interleukin, Tumor Necrosis Factor	Cytokines produced by the innate immune system that mostly cause inflammation while only few cytokines (like IL-10 and TRGF-b) are anti-inflammatory. The immune system maintains an equilibrium between pro- and anti-inflammatory signals to ensure a robust immune response without becoming self-destructive.
LF-EMFs	Low-Frequency Electromagnetic Fields	EMFs with frequencies below 300 kHz, radio wave frequency and below, containing insufficient energy to change chemical bonds causing, e.g., DNA damage.
ICNIRP	International Commission on Non-Ionizing Radiation Protection	ICNIRP continuously reviews scientific publications on the effects of EMF exposure on health and advises the WHO (World Health Organization) and the EU (European Union) on setting safety standards.

## Data Availability

No new data were created or analyzed in this study. Data sharing is not applicable to this article.

## References

[1] Finlay B.B., McFadden G. (2006). Anti-immunology: Evasion of the host immune system by bacterial and viral pathogens. Cell.

[2] Bowie A.G., Unterholzner L. (2008). Viral evasion and subversion of pattern-recognition receptor signaling. Nat. Rev. Immunol..

[3] Schneider W.M., Chevillotte M.D., Rice C.M. (2014). Interferon-stimulated genes: A complex web of host defenses. Annu. Rev. Immunol..

[4] McMichael A.J., Phillips R.E. (1997). Escape of human immunodeficiency virus from immune control. Annu. Rev. Immunol..

[5] Kwong P.D., Mascola J.R., Nabel G.J. (2013). Broadly neutralizing antibodies and the search for an HIV-1 vaccine: The end of the beginning. Nat. Rev. Immunol..

[6] Daugherty M.D., Malik H.S. (2012). Rules of engagement: Molecular insights from host-virus arms races. Annu. Rev. Gen..

[7] Vance R.E., Isberg R.R., Portnoy D.A. (2009). Patterns of Pathogenesis: Discrimination of Pathogenic and Nonpathogenic Microbes by the Innate Immune System. Cell Host Microbe.

[8] Bäumler A.J., Sperandio V. (2016). Interactions between the microbiota and pathogenic bacteria in the gut. Nature.

[9] Lambris J.D., Ricklin D., Geisbrecht B.V. (2008). Complement evasion by human pathogens. Microbiol. Nat. Rev. Microbiol..

[10] Donlan R.M. (2002). Biofilms: Microbial life on surfaces. Emerg. Infect. Dis..

[11] Soucy S.M., Huang J., Gogarten J.P. (2015). Horizontal gene transfer: Building the web of life. Nat. Rev. Gen..

[12] Good B.H., Bhatt A.S., McDonald M.J. (2025). Unraveling the tempo and mode of horizontal gene transfer in bacteria. Microbiol. Trends Microbiol..

[13] Zipfel P.F., Würzner R., Skerka C. (2007). Complement evasion of pathogens: Common strategies are shared by diverse organisms. Mol. Immunol..

[14] Zipfel P.F., Hallström T., Riesbeck K. (2013). Human complement control and complement evasion by pathogenic microbes—Tipping the balance. Mol. Immunol..

[15] Kopp E., Medzhitov R. (2003). Recognition of microbial infection by Toll-like receptors. Curr. Opin. Immunol..

[16] Girgis N.M., Dehaven B.C., Xiao Y., Alexander E., Viner K.M., Isaacs S.N. (2011). The Vaccinia virus complement control protein modulates adaptive immune responses during infection. J. Virol..

[17] Schenten D., Medzhitov R. (2011). The control of adaptive immune responses by the innate immune system. Adv. Immunol..

[18] Ploegh H.L. (1998). Viral strategies of immune evasion. Science.

[19] Beachboard D.C., Horner S.M. (2016). Innate immune evasion strategies of DNA and RNA viruses. Curr. Opin. Microbiol..

[20] Fensterl V., Sen G.C. (2009). Interferons and viral infections. BioFactors.

[21] Iwasaki A., Medzhitov R. (2015). Control of adaptive immunity by the innate immune system. Nat. Immunol..

[22] Schoggins J.W., Rice C.M. (2011). Interferon-stimulated genes and their antiviral effector functions. Curr. Opin. Virol..

[23] Ng C.T., Sullivan B.M., Teijaro J.R., Lee A.M., Welch M., Rice S., Sheehan K.C.F., Schreiber R.D., Oldstone M.B.A. (2015). Blockade of interferon Beta, but not interferon alpha, signaling controls persistent viral infection. Cell Host Microbe.

[24] Papayannopoulos V. (2018). Neutrophil extracellular traps in immunity and disease. Nat. Rev. Immunol..

[25] Jenne C.N., Wong C.H.Y., Zemp F.J., McDonald B., Rahman M.M., Forsyth P.A., McFadden G., Kubes P. (2013). Neutrophils recruited to sites of infection protect from virus challenge by releasing neutrophil extracellular traps. Cell Host Microbe.

[26] Pall M.L. (2013). Electromagnetic fields act via activation of voltage-gated calcium channels to produce beneficial or adverse effects. J. Cell. Mol. Med..

[27] Simko M. (2007). Cell Type Specific Redox Status is Responsible for Diverse Electromagnetic Field Effects. Curr. Med. Chem..

[28] Di Carlo A.L., White N.C., Litovitz T.A. (2001). Mechanical and electromagnetic induction of protection against oxidative stress. Bioelectrochemistry.

[29] McNab F., Mayer-Barber K., Sher A., Wack A., O’Garra A. (2015). Type I interferons in infectious disease. Nat. Rev. Immunol..

[30] Tecchio C., Cassatella M.A. (2016). Neutrophil-derived chemokines on the road to immunity. Sem. Immunol..

[31] Ivashkiv L.B., Donlin L.T. (2014). Regulation of type I interferon responses. Nat. Rev. Immunol..

[32] Simkó M., Mattsson M.-O. (2004). Extremely low frequency electromagnetic fields as effectors of cellular responses in vitro: Possible immune cell activation. J. Cell. Biochem..

[33] Scheiermann C., Kunisaki Y., Frenette P.S. (2013). Circadian control of the immune system. Nat. Rev. Immunol..

[34] Manzella N., Bracci M., Ciarapica V., Staffolani S., Strafella E., Rapisarda V., Valentino M., Amati M., Copertaro A., Santarelli L. (2015). Circadian gene expression and extremely low-frequency magnetic fields: An in vitro study. Bioelectromagnetics.

[35] Hartenstein V. (2006). Blood cells and blood cell development in the animal kingdom. Annu. Rev. Cell Dev..

[36] Kobayashi S.D., Malachowa N., DeLeo F.R. (2017). Influence of Microbes on Neutrophil Life and Death. Microbiol. Front. Cell. Infect. Microbiol..

[37] Kobayashi S.D., DeLeo F.R. (2009). Role of neutrophils in innate immunity: A systems biology-level approach. Wiley Interdiscip. Rev. Syst. Biol. Med..

[38] Netea M.G., Schlitzer A., Placek K., Joosten L.A.B., Schultze J.L. (2019). Innate and Adaptive Immune Memory: An Evolutionary Continuum in the Host’s Response to Pathogens. Cell Host Microbe.

[39] Masenga S.K., Mweene B.C., Luwaya E., Muchaili L., Chona M., Kirabo A. (2023). HIV–Host Cell Interactions. Cells.

[40] Le Negrate G. (2012). Viral interference with innate immunity by preventing NF-κB activity. Microbiol. Cell. Microbiol..

[41] Kikkert M. (2020). Innate Immune Evasion by Human Respiratory RNA Viruses. J. Innate Immun..

[42] Khan N., Vidyarthi A., Javed S., Agrewala J.N. (2016). Innate Immunity Holding the Flanks until Reinforced by Adaptive Immunity against Mycobacterium tuberculosis Infection. Front. Microbiol..

[43] Olson P.D., Hunstad D.A. (2016). Subversion of Host Innate Immunity by Uropathogenic Escherichia coli. Pathogens.

[44] Johannessen M., Askarian F., Sangvik M., Sollid J.E. (2013). Bacterial interference with canonical NFκB signalling. Microbiology.

[45] Askarian F., Wagner T., Johannessen M., Nizet V. (2018). *Staphylococcus aureus* modulation of innate immune responses through Toll-like (TLR), (NOD)-like (NLR) and C-type lectin (CLR) receptors. FEMS Microbiol. Rev..

[46] Tan A., Alsenani Q., Lanz M., Birchall C., Drage L.K.L., Picton D., Mowbray C., Ali A., Harding C., Pickard R.S. (2023). Evasion of toll-like receptor recognition by *Escherichia coli* is mediated via population level regulation of flagellin production. Front. Microbiol..

[47] Triboulet S., Subtil A. (2019). Make It a Sweet Home: Responses of Chlamydia trachomatis to the Challenges of an Intravacuolar Lifestyle. Microbiol. Spectrum.

[48] Phillip N.J., Zwack E.E., Brodsky I.E., Backert S. (2016). Activation and evasion of inflammasomes by *Yersinia*. Inflammasome Signalling and Bacterial Infections.

[49] Pamer E.G. (2004). Immune responses to *Listeria monocytogenes*. Nat. Rev. Immunol..

[50] Hodgins D.C., Kulkarni R.R., Shewen P.E. (2022). Subversion of the Immune Response by Bacterial Pathogens. Pathogenesis of Bacterial Infections in Animals.

[51] Cornelis G.R. (2002). The Yersinia Ysc-Yop “type III” weaponry. Mol. Cell Nat. Rev. Mol. Cell Biol..

[52] Brodsky I.E., Palm N.W., Sadanand S., Ryndak M.B., Sutterwala F.S., Flavell R.A., Bliska J.B., Medzhitov R. (2010). A Yersinia effector protein promotes virulence by preventing inflammasome recognition of the type III secretion system. Cell Host Microbe.

[53] Ashida H., Mimuro H., Sasakawa C. (2015). *Shigella* manipulates host immune responses by delivering effector proteins with specific roles. Front. Immunol..

[54] Spaan A.N., Surewaard B.G.J., Nijland R., van Strijp J.A.G. (2013). Neutrophils versus *Staphylococcus aureus*: A biological tug of war. Annu. Rev. Microbiol..

[55] Rooijakkers S.H.M., van Strijp J.A.G. (2007). Bacterial complement evasion. Mol. Immunol..

[56] García-Sastre A. (2001). Inhibition of interferon-mediated antiviral responses by influenza A viruses and other negative-strand RNA viruses. Virology.

[57] Ramachandran A., Horvath C.M. (2009). Paramyxovirus disruption of interferon signal transduction: STATus report. J. Interferon Cytokine Res..

[58] Lei X., Dong X., Ma R., Wang W., Xiao X., Tian Z., Wang C., Wang Y., Li L., Ren L. (2020). Activation and evasion of type I interferon responses by SARS-CoV-2. Nat. Commun..

[59] Fredericksen B.L., Keller B.C., Fornek J., Katze M.G., Gale M. (2008). Establishment and maintenance of the innate antiviral response to West Nile Virus involves both RIG-I and MDA5 signaling through IPS-1. J. Virol..

[60] Bekkering S., Torensma R. (2013). Another look at the life of a neutrophil. World J. Hematol..

[61] Koenderman L., Vrisekoop N. (2024). Extramedullary neutrophil progenitors: Quo Vadis?. Cell Mol. Immunol..

[62] Marković D., Maslovarić I., Djikić D., Čokić V.P. (2022). Neutrophil Death in Myeloproliferative Neoplasms: Shedding More Light on Neutrophils as a Pathogenic Link to Chronic Inflammation. Int. J. Mol. Sci..

[63] Eash K.J., Greenbaum A.M., Gopalan P.K., Link D.C. (2010). CXCR2 and CXCR4 antagonistically regulate neutrophil trafficking from murine bone marrow. J. Clin. Investig..

[64] Nourshargh S., Alon R. (2014). Leukocyte migration into inflamed tissues. Immunity.

[65] Martin C., Burdon P.C.E., Bridger G., Gutierrez-Ramos J.C., Williams T.J., Rankin S.M. (2003). Chemokines acting via CXCR2 and CXCR4 control the release of neutrophils from the bone marrow and their return following senescence. Immunity.

[66] Németh T., Mócsai A. (2016). Feedback Amplification of Neutrophil Function. Trends Immunol..

[67] Francis A., Bosio E., Stone S.F., Fatovich D.M., Arendts G., MacDonald S.P.J., Burrows S., Brown S.G.A. (2019). Markers Involved in Innate Immunity and Neutrophil Activation are Elevated during Acute Human Anaphylaxis: Validation of a Microarray Study. J. Innate Immun..

[68] Grieshaber-Bouyer R., Nigrovic P.A. (2019). Neutrophil Heterogeneity as Therapeutic Opportunity in Immune-Mediated Disease. Front. Immunol..

[69] Christoffersson G., Phillipson M. (2018). The neutrophil: One cell on many missions or many cells with different agendas?. Cell Tissue Res..

[70] Sollberger G., Brenes A.J., Warner J., Arthur J.S.C., Howden A.J.M. (2024). Quantitative proteomics reveals tissue-specific, infection-induced and species-specific neutrophil protein signatures. Sci. Rep..

[71] Strydom N., Rankin S.M. (2013). Regulation of circulating neutrophil numbers under homeostasis and in disease. J. Innate Immun..

[72] Summers C., Rankin S.M., Condliffe A.M., Singh N., Peters A.M., Chilvers E.R. (2010). Neutrophil kinetics in health and disease. Trends Immunol..

[73] Kawai T., Akira S. (2010). The role of pattern-recognition receptors in innate immunity: Update on Toll-like receptors. Nat. Immunol..

[74] Takeda K., Kaisho T., Akira S. (2003). Toll-like receptors. Annu. Rev. Immunol..

[75] Loo Y.-M., Gale M. (2011). Immune signaling by RIG-I-like receptors. Immunity.

[76] Philpott D.J., Girardin S.E. (2010). Nod-like receptors: Sentinels at host membranes. Curr. Opin. Immunol..

[77] Schroder K., Tschopp J. (2010). The inflammasomes. Cell.

[78] Mayadas T.N., Cullere X., Lowell C.A. (2014). The multifaceted functions of neutrophils. Annu. Rev. Pathol..

[79] Ricklin D., Hajishengallis G., Yang K., Lambris J.D. (2010). Complement: A key system for immune surveillance and homeostasis. Nat. Immunol..

[80] Reis e Sousa C., Yamasaki S., Brown G.D. (2024). Myeloid C-type lectin receptors in innate immune recognition. Immunity.

[81] Mol S., Hafkamp F.M.J., Varela L., Simkhada N., Taanman-Kueter E.W., Tas S.W., Wauben M.H.M., Groot Kormelink T., de Jong E.C. (2021). Efficient Neutrophil Activation Requires Two Simultaneous Activating Stimuli. Int. J. Mol. Sci..

[82] Nauseef W.M., Borregaard N. (2014). Neutrophils at work. Nat. Immunol..

[83] Lacy P. (2006). Mechanisms of degranulation in neutrophils. Allergy Asthma Clin. Immunol..

[84] Kolaczkowska E., Kubes P. (2013). Neutrophil recruitment and function in health and inflammation. Nat. Rev. Immunol..

[85] Rosales C. (2018). Neutrophil: A Cell with Many Roles in Inflammation or Several Cell Types?. Front. Physiol..

[86] Burn G.L., Foti A., Marsman G., Patel D.F., Zychlinsky A. (2021). The Neutrophil. Immunity.

[87] Sollberger G., Tilley D.O., Zychlinsky A. (2018). Neutrophil Extracellular Traps: The Biology of Chromatin Externalization. Dev. Cell.

[88] Borregaard N., Herlin T. (1982). Energy metabolism of human neutrophils during phagocytosis. J. Clin. Investig..

[89] Mantovani A., Cassatella M.A., Costantini C., Jaillon S. (2011). Neutrophils in the activation and regulation of innate and adaptive immunity. Nat. Rev. Immunol..

[90] Silvestre-Roig C., Fridlender Z.G., Glogauer M., Scapini P. (2019). Neutrophil Diversity in Health and Disease. Trends Immunol..

[91] Shafqat A., Khan J.A., Alkachem A.Y., Sabur H., Alkattan K., Yaqinuddin A., Sing G.K. (2023). How Neutrophils Shape the Immune Response: Reassessing Their Multifaceted Role in Health and Disease. Int. J. Mol. Sci..

[92] Bratton D.L., Henson P.M. (2011). Neutrophil clearance: When the party is over, clean-up begins. Trends Immunol..

[93] Sweet M.J., Ramnath D., Singhal A., Kapetanovic R. (2025). Inducible antibacterial responses in macrophages. Nat. Rev. Immunol..

[94] Uribe-Querol E., Rosales C. (2020). Phagocytosis: Our Current Understanding of a Universal Biological Process. Front. Immunol..

[95] Kovacs S.B., Miao E.A. (2017). Gasdermins: Effectors of Pyroptosis. Trends Cell Biol..

[96] Yipp B.G., Petri B., Salina D., Jenne C.N., Scott B.N.V., Zbytnuik L.D., Pittman K., Asaduzzaman M., Wu K., Meijndert H.C. (2012). Infection-induced NETosis is a dynamic process involving neutrophil multitasking in vivo. Nat. Med..

[97] Pilsczek F.H., Salina D., Poon K.K.H., Fahey C., Yipp B.G., Sibley C.D., Robbins S.M., Green F.H.Y., Surette M.G., Sugai M. (2010). A novel mechanism of rapid nuclear neutrophil extracellular trap formation in response to *Staphylococcus aureus*. J. Immunol..

[98] Remijsen Q., Berghe T.V., Wirawan E., Asselbergh B., Parthoens E., De Rycke R., Noppen S., Delforge M., Willems J., Vandenabeele P. (2011). Neutrophil extracellular trap cell death requires both autophagy and superoxide generation. Cell Res..

[99] Vorobjeva N.V., Chernyak B.V. (2020). NETosis: Molecular Mechanisms, Role in Physiology and Pathology. Biochem. Biokhimiia.

[100] Pelletier M., Micheletti A., Cassatella M.A. (2010). Modulation of human neutrophil survival and antigen expression by activated CD4^+^ and CD8^+^ T cells. J. Leukoc. Biol..

[101] Rosales C., Demaurex N., Lowell C.A., Uribe-Querol E. (2016). Neutrophils: Their Role in Innate and Adaptive Immunity. J. Immunol. Res..

[102] Beauvillain C., Delneste Y., Scotet M., Peres A., Gascan H., Guermonprez P., Barnaba V., Jeannin P. (2007). Neutrophils efficiently cross-prime naive T cells in vivo. Blood.

[103] Abi Abdallah D.S., Egan C.E., Butcher B.A., Denkers E.Y. (2011). Mouse neutrophils are professional antigen-presenting cells programmed to instruct Th1 and Th17 T-cell differentiation. Int. Immunol..

[104] Moffat A., Gwyer Findlay E. (2024). Evidence for antigen presentation by human neutrophils. Blood.

[105] Vono M., Lin A., Norrby-Teglund A., Koup R.A., Liang F., Loré K. (2017). Neutrophils acquire the capacity for antigen presentation to memory CD4^+^ T cells in vitro and ex vivo. Blood.

[106] Branzk N., Papayannopoulos V. (2013). Molecular mechanisms regulating NETosis in infection and disease. Semin. Immunopathol..

[107] Puga I., Cols M., Barra C.M., He B., Cassis L., Gentile M., Comerma L., Chorny A., Shan M., Xu W. (2012). B cell–helper neutrophils stimulate the diversification and production of immunoglobulin in the marginal zone of the spleen. Nat. Immunol..

[108] Leliefeld P.H.C., Wessels C.M., Leenen L.P.H., Koenderman L., Pillay J. (2016). The role of neutrophils in immune dysfunction during severe inflammation. Crit. Care.

[109] Muñoz Carrillo J.L., Castro García F.P., Coronado O.G., Moreno García M.A., Contreras Cordero J.F., Rezaei N. (2017). Physiology and Pathology of Innate Immune Response Against Pathogens. Physiology and Pathology of Immunology.

[110] Lazear H.M., Schoggins J.W., Diamond M.S. (2019). Shared and Distinct Functions of Type I and Type III Interferons. Immunity.

[111] Ravindra N.G., Alfajaro M.M., Gasque V., Huston N.C., Wan H., Szigeti-Buck K., Yasumoto Y., Greaney A.M., Habet V., Chow R.D. (2021). Single-cell longitudinal analysis of SARS-CoV-2 infection in human airway epithelium identifies target cells, alterations in gene expression, and cell state changes. PLoS Biol..

[112] Blanco-Melo D., Nilsson-Payant B.E., Liu W.-C., Uhl S., Hoagland D., Møller R., Jordan T.X., Oishi K., Panis M., Sachs D. (2020). Imbalanced host response to SARS-CoV-2 drives development of COVID-19. Cell.

[113] Martin-Sancho L., Lewinski M.K., Pache L., Stoneham C.A., Yin X., Becker M.E., Pratt D., Churas C., Rosenthal S.B., Liu S. (2021). Functional landscape of SARS-CoV-2 cellular restriction. Mol. Cell.

[114] Hermesh T., Moltedo B., López C.B., Moran T.M. (2010). Buying time-the immune system determinants of the incubation period to respiratory viruses. Viruses.

[115] Severa M., Diotti R.A., Etna M.P., Rizzo F., Fiore S., Ricci D., Iannetta M., Sinigaglia A., Lodi A., Mancini N. (2021). Differential plasmacytoid dendritic cell phenotype and type I Interferon response in asymptomatic and severe COVID-19 infection. PLoS Pathog..

[116] Li S., Wu J., Zhu S., Liu Y.-J., Chen J. (2017). Disease-Associated Plasmacytoid Dendritic Cells. Front. Immunol..

[117] Pierce C.A., Sy S., Galen B., Goldstein D.Y., Orner E., Keller M.J., Herold K.C., Herold B.C. (2021). Natural mucosal barriers and COVID-19 in children. JCI Insight.

[118] Paget C., Doz-Deblauwe E., Winter N., Briard B. (2022). Specific NLRP3 Inflammasome Assembling and Regulation in Neutrophils: Relevance in Inflammatory and Infectious Diseases. Cells.

[119] Kelley N., Jeltema D., Duan Y., He Y. (2019). The NLRP3 Inflammasome: An Overview of Mechanisms of Activation and Regulation. Int. J. Mol. Sci..

[120] Pylaeva E., Lang S., Jablonska J. (2016). The Essential Role of Type I Interferons in Differentiation and Activation of Tumor-Associated Neutrophils. Front. Immunol..

[121] Loske J., Röhmel J., Lukassen S., Stricker S., Magalhães V.G., Liebig J., Chua R.L., Thürmann L., Messingschlager M., Seegebarth A. (2022). Pre-activated antiviral innate immunity in the upper airways controls early SARS-CoV-2 infection in children. Nat. Biotechnol..

[122] Chou J., Thomas P.G., Randolph A.G. (2022). Immunology of SARS-CoV-2 infection in children. Nat. Immunol..

[123] Schoggins J.W. (2019). Interferon-Stimulated Genes: What Do They All Do?. Annu. Rev. Virol..

[124] Hadjadj J., Yatim N., Barnabei L., Corneau A., Boussier J., Smith N., Péré H., Charbit B., Bondet V., Chenevier-Gobeaux C. (2020). Impaired type I interferon activity and inflammatory responses in severe COVID-19 patients. Science.

[125] Feld J.J., Kandel C., Biondi M.J., A Kozak R., Zahoor M.A., Lemieux C., Borgia S.M., Boggild A.K., Powis J., McCready J. (2021). Peginterferon lambda for the treatment of outpatients with COVID-19: A phase 2, placebo-controlled randomised trial. Lancet Resp. Med..

[126] Galani I.-E., Rovina N., Lampropoulou V., Triantafyllia V., Manioudaki M., Pavlos E., Koukaki E., Fragkou P.C., Panou V., Rapti V. (2021). Untuned antiviral immunity in COVID-19 revealed by temporal type I/III interferon patterns and flu comparison. Nat. Immunol..

[127] Wang N., Zhan Y., Zhu L., Hou Z., Liu F., Song P., Qiu F., Wang X., Zou X., Wan D. (2020). Retrospective Multicenter Cohort Study Shows Early Interferon Therapy Is Associated with Favorable Clinical Responses in COVID-19 Patients. Cell Host Microbe.

[128] Golbach L.A., Scheer M.H., Cuppen J.J.M., Savelkoul H., Verburg-van Kemenade B.M.L. (2015). Low-Frequency Electromagnetic Field Exposure Enhances Extracellular Trap Formation by Human Neutrophils through the NADPH Pathway. J. Innate Immun..

[129] Cuppen J.J.M., Gradinaru C., Raap-van Sleuwen B.E., de Wit A.C.E., van der Vegt T.A.A.J., Savelkoul H.F.J. (2022). LF-EMF Compound Block Type Signal Activates Human Neutrophilic Granulocytes In Vivo. Bioelectromagnetics.

[130] Channappanavar R., Fehr A.R., Zheng J., Wohlford-Lenane C., Abrahante J.E., Mack M., Sompallae R., McCray P.B., Meyerholz D.K., Perlman S. (2019). IFN-I response timing relative to virus replication determines MERS coronavirus infection outcomes. J. Clin. Investig..

[131] Abraham S.N., Miao Y. (2015). The nature of immune responses to urinary tract infections. Nat. Rev. Immunol..

[132] Herant M., Marganski W.A., Dembo M. (2003). The mechanics of neutrophils: Synthetic modeling of three experiments. Biophys. J..

[133] Herant M., Heinrich V., Dembo M. (2006). Mechanics of neutrophil phagocytosis: Experiments and quantitative models. J. Cell Sci..

[134] Jesaitis A.J., Franklin M.J., Berglund D., Sasaki M., Lord C.I., Bleazard J.B., Duffy J.E., Beyenal H., Lewandowski Z. (2003). Compromised host defense on Pseudomonas aeruginosa biofilms: Characterization of neutrophil and biofilm interactions. J. Immunol..

[135] van der Poll T., Marchant A., Keogh C.V., Goldman M., Lowry S.F. (1996). Interleukin-10 impairs host defense in murine pneumococcal pneumonia. J. Infect. Dis..

[136] Brinkmann V., Reichard U., Goosmann C., Fauler B., Uhlemann Y., Weiss D.S., Weinrauch Y., Zychlinsky A. (2004). Neutrophil extracellular traps kill bacteria. Science.

[137] Riaz B., Sohn S. (2023). Neutrophils in Inflammatory Diseases: Unraveling the Impact of Their Derived Molecules and Heterogeneity. Cells.

[138] Monem S., Furmanek-Blaszk B., Łupkowska A., Kuczynska-Wisnik D., Stojowska-Swedrzynska K., LaskowskaInt E. (2020). Mechanisms Protecting Acinetobacter baumannii against Multiple Stresses Triggered by the Host Immune Response, Antibiotics and Outside-Host Environment. J. Mol. Sci..

[139] Jeffreys S., Chambers J.P., Yu J.-J., Hung C.-Y., Forsthuber T., Arulanandam B.P. (2022). Insights into Acinetobacter baumannii protective immunity. Front. Immunol..

[140] Elbim C., Katsikis P.D., Estaquier J. (2009). Neutrophil apoptosis during viral infections. Open Virol. J..

[141] Salamone G.V., Petracca Y., Fuxman Bass J.I., Rumbo M., Nahmod K.A., Gabelloni M.L., Vermeulen M.E., Matteo M.J., Geffner J.R., Trevani A.S. (2010). Flagellin delays spontaneous human neutrophil apoptosis. Lab. Investig..

[142] Kobayashi S.D., Braughton K.R., Whitney A.R., Voyich J.M., Schwan T.G., Musser J.M., DeLeo F.R. (2003). Bacterial pathogens modulate an apoptosis differentiation program in human neutrophils. Proc. Natl. Acad. Sci. USA.

[143] Janssen L., Muller H.S., Martins V.d.P. (2022). Unweaving the NET: Microbial strategies for neutrophil extracellular trap evasion. Microb. Pathog..

[144] Baz A.A., Hao H., Lan S., Li Z., Liu S., Chen S., Chu Y. (2024). Neutrophil extracellular traps in bacterial infections and evasion strategies. Front. Immunol..

[145] Kobayashi S.D., Malachowa N., DeLeo F.R. (2018). Neutrophils and Bacterial Immune Evasion. J. Innate Immun..

[146] Urban C.F., Lourido S., Zychlinsky A. (2006). How do microbes evade neutrophil killing?. Microbiol. Cell. Microbiol..

[147] Schönrich G., Raftery M.J. (2016). Neutrophil Extracellular Traps Go Viral. Front. Immunol..

[148] Chen J., He R., Luo J., Yan S., Zhu W., Liu S. (2025). Neutrophil Extracellular Traps in Viral Infections. Pathogens.

[149] Johansson C., Kirsebom F.C.M. (2021). Neutrophils in respiratory viral infections. Mucosal Immunol..

[150] Rawat S., Vrati S., Banerjee A. (2021). Neutrophils at the crossroads of acute viral infections and severity. Mol. Asp. Med..

[151] Kruger P., Saffarzadeh M., Weber A.N.R., Rieber N., Radsak M., von Bernuth H., Benarafa C., Roos D., Skokowa J., Hartl D. (2015). Neutrophils: Between host defence, immune modulation, and tissue injury. PLoS Pathog..

[152] Zhang F., Xia Y., Su J., Quan F., Zhou H., Li Q., Feng Q., Lin C., Wang D., Jiang Z. (2024). Neutrophil diversity and function in health and disease. Signal Transduct. Target. Ther..

[153] Tang X.-D., Ji T.-T., Dong J.-R., Feng H., Chen F.-Q., Chen X., Zhao H.-Y., Chen D.-K., Ma W.-T. (2021). Pathogenesis and Treatment of Cytokine Storm Induced by Infectious Diseases. Int. J. Mol. Sci..

[154] Mortaz E., Alipoor S.D., Adcock I.M., Mumby S., Koenderman L. (2018). Update on Neutrophil Function in Severe Inflammation. Front. Immunol..

[155] Honda T., Uehara T., Matsumoto G., Arai S., Sugano M. (2016). Neutrophil left shift and white blood cell count as markers of bacterial infection. Clin. Chim. Acta.

[156] Carissimo G., Xu W., Kwok I., Abdad M.Y., Chan Y.-H., Fong S.-W., Puan K.J., Lee C.Y.-P., Yeo N.K.-W., Amrun S.N. (2020). Whole blood immunophenotyping uncovers immature neutrophil-to-VD2 T-cell ratio as an early marker for severe COVID-19. Nat. Commun..

[157] Zini G., Bellesi S., Ramundo F., d’Onofrio G. (2020). Morphological anomalies of circulating blood cells in COVID-19. Am. J. Hematol..

[158] Hiti L., Markovič T., Lainscak M., Farkaš Lainščak J., Pal E., Mlinarič-Raščan I. (2025). The immunopathogenesis of a cytokine storm: The key mechanisms underlying severe COVID-19. Cytokine Growth Factor Rev..

[159] Morrissey S.M., Geller A.E., Hu X., Tieri D., Ding C., Klaes C.K., Cooke E.A., Woeste M.R., Martin Z.C., Chen O. (2021). A specific low-density neutrophil population correlates with hypercoagulation and disease severity in hospitalized COVID-19 patients. JCI Insight.

[160] Wang J., Chen L., Wu Y. (2022). Advances in the mechanism of bacterial escape neutrophil killing. Chin. J. Cell. Mol. Immunol..

[161] Qin C., Zhou L., Hu Z., Zhang S., Yang S., Tao Y., Xie C., Ma K., Shang K., Wang W. (2020). Dysregulation of immune response in patients with Coronavirus 2019 (COVID-19) in Wuhan, China. Clin. Infect. Dis..

[162] Jones E., Sheng J., Carlson J., Wang S. (2021). Aging-induced fragility of the immune system. J. Theor. Biol..

[163] Fulop T., Larbi A., Dupuis G., Le Page A., Frost E.H., Cohen A.A., Witkowski J.M., Franceschi C. (2018). Immunosenescence and inflammaging as two sides of the same coin: Friends or foes?. Front. Immunol..

[164] Fulop T., Page A.L., Fortin C., Witkowski J.M., Dupuis G., Larbi A. (2014). Cellular signaling in the aging immune system. Curr. Opin. Immunol..

[165] Kapri-Pardes E., Hanoch T., Maik-Rachline G., Murbach M., Bounds P.L., Kuster N., Seger R. (2017). Activation of Signaling Cascades by Weak Extremely Low Frequency Electromagnetic Fields. Cell. Physiol. Biochem..

[166] Mahaki H., Tanzadehpanah H., Jabarivasal N., Sardanian K., Zamani A. (2019). A review on the effects of extremely low frequency electromagnetic foeld (ELF-EMF) in cytokines of innate and adaptive immunity. Electromagn. Biol. Med..

[167] Poniedzialek B., Rzymski P., Nawrocka-Bogusz H., Jaroszyk F., Wiktorowicz K. (2013). The effect of electromagnetic field on reactive oxygen species production in human neutrophils in vitro. Electromagn. Biol. Med..

[168] ICNIRP (2020). Guidelines for limiting exposure to electromagnetic fields (100 kHz–300 GHz). Health Phys..

[169] Golbach L.A., Philippi J.G.M., Cuppen J.J.M., Savelkoul H.F.J., Verburg-van Kemenade B.M.L. (2015). Calcium signalling in human neutrophil cell lines is not affected by low-frequency electromagnetic fields. Bioelectromagnetics.

[170] Osera C., Amadio M., Falone S., Fassina L., Magenes G., Amicarelli F., Ricevuti G., Govoni S., Pascale A. (2015). Pre-exposure of neuroblastoma cell line to pulsed electromagnetic field prevents H_2_O_2_-induced ROS production by increasing MnSOD activity. Bioelectromagnetics.

[171] Hiscock H.G., Worster S., Kattnig D.R., Steers C., Jin Y., Manolopoulos D.E., Mouritsen H., Hore P.J. (2016). *The quantum* needle of the avian magnetic compass. Proc. Natl. Acad. Sci. USA.

[172] Rosado M.M., Simkó M., Mattsson M.-O., Pioli C. (2018). Immune-Modulating Perspectives for Low Frequency Electromagnetic Fields in Innate Immunity. Front. Public Health.

[173] Thomas A.W., Graham K., Prato F.S., McKay J., Forster P.M., Moulin D.E., Chari S. (2007). A Randomized, Double-Blind, Placebo-Controlled Clinical Trial Using a Low-Frequency Magnetic Field in the Treatment of Musculoskeletal Chronic Pain. Pain Res. Manag..

[174] Jing D., Zhai M., Tong S., Xu F., Cai J., Shen G., Wu Y., Li X., Xie K., Liu J. (2016). Pulsed electromagnetic fields promote osteogenesis and osseointegration of porous titanium implants in bone defect repair through a Wnt/β-catenin signaling-associated mechanism. Sci. Rep..

[175] Saliev T., Mustapova Z., Kulsharova G., Bulanin D., Mikhalovsky S. (2014). Therapeutic potential of electromagnetic fields for tissue engineering and wound healing. Cell Prolif..

[176] Ross C.L., Ang D.C., Almeida-Porada G. (2019). Targeting Mesenchymal Stromal Cells/Pericytes (MSCs) With Pulsed Electromagnetic Field (PEMF) Has the Potential to Treat Rheumatoid Arthritis. Front. Immunol..

[177] Elmusharaf M.A., Cuppen J.J., Grooten H.N., Beynen A.C. (2007). Antagonistic effect of electromagnetic field exposure on coccidiosis infection in broiler chickens. Poultry Sci..

[178] Cuppen J.J.M., Wiegertjes G.F., Lobee H.W.J., Savelkoul H.F.J., Elmusharaf M.A., Beynen A.C., Grooten H.N.A., Smink W. (2007). Immune stimulation in fish and chicken through weak low frequency electromagnetic fields. Environmentalist.

[179] Rosenspire A.J., Kindzelskii A.L., Simon B.J., Petty H.R. (2005). Real-time control of neutrophil metabolism by very weak ultra-low frequency pulsed magnetic fields. Biophys. J..

[180] Linnemann C., Sahin F., Chen Y., Falldorf K., Ronniger M., Histing T., Nussler A.K., Ehnert S. (2023). NET Formation Was Reduced via Exposure to Extremely Low-Frequency Pulsed Electromagnetic Fields. Int. J. Mol. Sci..

[181] Vlasova I.I., Mikhalchik E.V., Gusev A.A., Balabushevich N.G., Gusev S.A., Kazarinov K.D. (2018). Extremely high-frequency electromagnetic radiation enhances neutrophil response to particulate agonists. Bioelectromagnetics.

[182] De Kleijn S., Ferwerda G., Wiese M., Trentelman J., Cuppen J., Kozicz T., de Jager L., Hermans P.W., Verburg-van Kemenade B.M. (2016). A short-term extremely low frequency electromagnetic field exposure increases circulating leukocyte numbers and affects HPA-axis signaling in mice. Bioelectromagnetics.

[183] Lei H., Pan Y., Wu R., Lv Y. (2020). Innate Immune Regulation Under Magnetic Fields With Possible Mechanisms and Therapeutic Applications. Front. Immunol..

[184] Segal A.W. (2005). How neutrophils kill microbes. Annu. Rev. Immunol..

[185] Esaki M., Okuya K., Tokorozaki K., Haraguchi Y., Hasegawa T., Ozawa M. (2025). Highly Pathogenic Avian Influenza A(H5N1) Outbreak in Endangered Cranes, Izumi Plain, Japan, 2022–2023. Emerg. Infect. Dis..

[186] Cassatella M.A., Östberg N.K., Tamassia N., Soehnlein O. (2019). Biological Roles of Neutrophil-Derived Granule Proteins and Cytokines. Trends Immunol..

[187] Paudel S., Ghimire L., Jin L., Jeansonne D., Jeyaseelan S. (2022). Regulation of emergency granulopoiesis during infection. Front. Immunol..

[188] Vergadi E., Kolliniati O., Lapi I., Ieronymaki E., Lyroni K., Alexaki V.I., Diamantaki E., Vaporidi K., Hatzidaki E., Papadaki H.A. (2024). An IL-10/DEL-1 axis supports granulopoiesis and survival from sepsis in early life. Nat. Commun..

[189] Drifte G., Dunn-Siegrist I., Tissières P., Pugin J. (2013). Innate immune functions of immature neutrophils in patients with sepsis and severe systemic inflammatory response syndrome. Crit. Care Med..

[190] Wu W., Zhang W., Tian L., Brown B.R., Walters M.S., Metcalf J.P. (2020). IRF7 Is Required for the Second Phase Interferon Induction during Influenza Virus Infection in Human Lung Epithelia. Viruses.

[191] Hartshorn K.L. (2020). Innate Immunity and Influenza A Virus Pathogenesis: Lessons for COVID-19. Microbiol. Front. Cell. Infect. Microbiol..

[192] Purbey P.K., Roy K., Gupta S., Manash K., Paul M.K. (2023). Mechanistic insight into the protective and pathogenic immune-responses against SARS-CoV-2. Mol. Immunol..

